# MERS and COVID‐19: A double burden for the healthcare system of Saudi Arabia

**DOI:** 10.1002/hsr2.515

**Published:** 2022-02-18

**Authors:** Rabia Waseem, Irfan Ullah, Muhammad Irfan, Asimina Dominari, Osman Kamal Osman Elmahi, Muhammad Junaid Tahir

**Affiliations:** ^1^ Karachi Medical and Dental College Karachi Pakistan; ^2^ Kabir Medical College, Gandhara University Peshawar Pakistan; ^3^ Internal Medicine Hayatabad Medical Complex Peshawar Pakistan; ^4^ School of Medicine Aristotle University of Thessaloniki Thessaloniki Greece; ^5^ Faculty of Medicine Ibn Sina University Khartoum Sudan; ^6^ Ameer‐ud‐Din Medical College, University of Health and Sciences Lahore Pakistan; ^7^ Lahore General Hospital Lahore Pakistan

To the Editor,

The severe acute respiratory syndrome coronavirus‐2 (SARS‐CoV‐2), and the Middle Eastern respiratory syndrome coronavirus (MERS‐CoV) are pathogens belonging to the family of zoonotic beta coronaviruses that cause infection in humans.^1^ The clinical presentation of both, coronavirus disease 2019 (COVID‐19) caused by SARS‐CoV‐2 and Middle Eastern respiratory syndrome (MERS) caused by MERS‐CoV, can vary from completely asymptomatic to the development of respiratory tract disease of varying severity, which can potentially be fatal.^1^ Saudi Arabia has been in the epicenter of the MERS outbreak since 2012, when the first case, that of an adult male who expired due to a severe lower respiratory tract infection, was officially reported.^2^ The first few cases of COVID‐19 were recognized in Wuhan, China, in January 2020 and its rapid worldwide spread led to the World Health Organization (WHO) declaration of the COVID‐19 pandemic on 11 March 2020.^1^ The latter then raised alarm among the public health authorities across the globe.^3^ Saudi Arabia reported its first case of COVID‐19 in March 2020,^3^ and its healthcare system has since then been facing the challenge of simultaneously keeping under control two potentially deadly viral infections. We herein attempt to shed light on the double burden currently faced by the healthcare system of Saudi Arabia, as well as stress the importance of concurrent management and prevention measures accounting for both COVID‐19 and MERS in this region.

As of 1 March 2021, 2586 cases and 939 deaths due to MERS have been reported worldwide.^4^ A study investigating the prevalence of MERS infection among camels in Saudi Arabia has shown that 90% of adult dromedary camels had been infected by the virus. These camels have likely served as an important reservoir for the spread of MERS‐CoV to humans. It is, therefore, not a surprise that, among the five MERS cases reported in Saudi Arabia from 1 January to 1 March 2022, three had mentioned contact with camels.^4^, ^5^ Moreover, although limited, there is still potential for secondary spread of MERS contracted from camels within the household, and secondary spread in the hospital setting is capable of causing bigger outbreaks.^6^ As a result of the concomitant spread of COVID‐19, the allocation of healthcare resources to both diseases may be problematic, and nosocomial spread of both viruses may pose a risk of infection of healthcare workers as well as their families and close contacts.

MERS‐CoV has a very high mortality rate (34.77%), much higher than SARS‐CoV‐2 (10.87%), but the latter is much more contagious.^1^ Among the aforementioned five MERS cases, unfortunately three have expired.^4^ MERS‐CoV presents with nonspecific symptoms in the early stages of the disease, making it difficult to detect.^7^ Asymptomatic infection, which is far more prevalent in SARS‐CoV‐2,^8^ is also a serious cause of concern, as it can silently lead to propagation of the infection. Both SARS‐CoV‐2 and MERS‐CoV cause more severe disease in adult males and those with comorbidities^8^; patients with congestive heart failure (CHF) and chronic kidney disease are also known to deteriorate much more rapidly.^9^, ^10^ The presenting symptoms of both pathogens are similar, including fever and cough, which may or may not progress to more severe manifestations, such as pneumonia.^11^ MERS and COVID‐19 exhibit similarities in terms of diagnosis and management.^9^ The many overlapping characteristics of these diseases make education of the susceptible population extremely critical, and they additionally pose a challenge upon the healthcare workers in the country to identify early and to differentiate among the two diseases, while taking adequate measures to secure their own safety. The question regarding a possible interaction between the two viruses also arises here, as they can both infect an individual if present in the same environment. However, there are less data available on the subject and a recent study revealed lack of concurrent MERS‐CoV and SARS‐CoV‐2 infection.^8^

Coronaviruses are known to have a seasonal variation; the outbreak of MERS‐CoV has been reported mainly in the summer period (May‐June), whereas infections from SARS‐CoV‐2 have a tendency to peak during the winter season.^1^ With summer getting closer, MERS cases are likely imminent. We could reasonably postulate that the association between the two infections could potentially cover the whole year. Furthermore, it is worth highlighting that unlike SARS‐CoV‐2, no vaccine for the MERS‐CoV has been developed yet.^12^ Although several drugs have been used to treat COVID‐19 and MERS patients, however, currently SARS‐CoV‐2 and MERS‐CoV have no specific antiviral treatment available and further research and therapeutic alternatives are still required.^13^, ^14^ Considering all these factors along with the rise of the transmission curve of COVID‐19 in Saudi Arabia,^15^ the situation needs to be handled with utmost urgency. Hence, adopting strict policies with regard to social distancing and smart working, as well as constantly educating the general public upon the topics of usage of masks, hand washing and importance of getting vaccinated (in the case of COVID‐19) can make a significant difference to help prevent and control infections. In addition, testing the population for MERS‐CoV alongside SARS‐CoV‐2 could potentially spare the country from the struggle of combating two overwhelming outbreaks at the same time.

As opposed to SARS‐CoV‐2, MERS‐CoV affects Saudi citizens more than non‐Saudi residents; however, these data are based on comparison between a single COVID‐19 outbreak to that of MERS‐CoV^9^ and further demographic studies with respect to the ongoing COVID‐19 pandemic may present with varying results. The population of Saudi Arabia exceeds 34 million, 63% being Saudi citizens. The majority of the population comprises adults between 15 and 64 years of age. Both MERS‐CoV and SARS‐CoV‐2 affect adults in this age group the most, thus making the Saudi population particularly vulnerable to both infections.^9^, ^16^ The epidemiological distribution of COVID‐19 and MERS during the last outbreaks was highest in the western region of Saudi Arabia.^9^ This region contains the cities of Makkah and Medina, both of which entertain thousands of Muslim pilgrims from all over the world to perform the activities of the once‐a‐year Hajj and the all‐year‐round Umrah. Two out of the five recent cases of MERS were identified in Makkah.^4^ This alarming situation calls for attention by the country's health authority to reevaluate the preventive measures with regards to travelling as well as case detection. In the light of these findings, the Kingdom needs to show equity in terms of providing special intervention according to the distribution of the disease, the population most vulnerable, and the presence of risk factors for the transmission of infection. In addition, more studies should be conducted regarding the epidemiology and surveillance for MERS, especially as cases continue to rise.

Despite the recent re‐allocation of healthcare resources to the treatment of COVID‐19 patients, specifically in areas at high risk for a MERS outbreak like Saudi Arabia, more attention should be paid to providing testing facilities for both SARS‐CoV‐2 and MERS‐CoV. In order to minimize the spread of MERS‐CoV via adult dromedary camels, healthcare authorities should devise a special health surveillance system for camel workers and others exposed to these animals. Targeted preventions and intervention in the form of physical distancing, smart lockdowns, and vaccinations should be administered to the population and areas more likely to be affected by the viruses. Furthermore, the conduction of more research focusing on viral interactions and risk of transmission will assist the healthcare workers to effectively protect themselves and control disease spread. Having experienced MERS‐CoV before, the Kingdom has made commendable attempts in controlling COVID‐19, especially through emphasizing the value of educating the general population through public awareness campaigns, which have led to better and safer practices.^17^ However, a lot still needs to be done to the direction of informing and educating the population, and adherence to infection prevention and control measures also within healthcare facilities is required in order to prevent further nosocomial and community outbreaks.

In conclusion, it is important to highlight that both SARS‐CoV2 and MERS‐CoV are deadly pathogens that can infect the Saudi population and deteriorate the Saudi healthcare system, especially in the case of a combined outbreak. Raising awareness among the masses regarding the severity of the current situation will lead to better practice of social distancing and maintenance of good hygiene. Alerting the country's healthcare authority to take timely measures in order to control infections from both of these pathogens will not only help to preserve its resources but will also lead to improvement of management and protocol of the diseases inside the hospital setting. Moreover, allocating healthcare resources toward accelerating the process of developing a vaccine to combat MERS, and carrying out more research in areas involving the pathogenesis and demographics of COVID‐19 and MERS, could potentially open doors toward further studies and aid in improving prevention measures and treatment.

## FUNDING

The authors declare that there are no funding sources for this paper.

## CONFLICT OF INTEREST

The authors declare that there is no conflict of interests.

## AUTHOR CONTRIBUTIONS

Conceptualization: Rabia Waseem, Irfan Ullah

Writing—original draft: Rabia Waseem, Irfan Ullah, Muhammad Irfan, Asimina Dominari, Osman Kamal Osman Elmahi

Writing—review and editing: Muhammad Junaid Tahir

All authors have read and agreed to the final version of the manuscript.
